# Engineering Single-Chain Antibody Fragment (scFv) Variants Targeting A Disintegrin and Metalloproteinase-17 (ADAM-17)

**DOI:** 10.3390/biom16010031

**Published:** 2025-12-24

**Authors:** Masoud Kalantar, Elham Khorasani Buxton, Korey M. Reid, Donald Bleyl, David M. Leitner, Maryam Raeeszadeh-Sarmazdeh

**Affiliations:** 1 Department of Chemical and Materials Engineering, University of Nevada, Reno, NV 89557, USA; 2 Department of Computer Science, University of Illinois, Springfield, IL 62703, USA; esahe2@uis.edu (E.K.B.); dbleyl3@gatech.edu (D.B.); 3 Department of Chemistry, University of Nevada, Reno, NV 89557, USA

**Keywords:** metalloproteinase, antibody engineering, ADAM-17, directed evolution, single-chain variable fragment (scFv), yeast surface display, engineering protease inhibitors

## Abstract

Metalloproteinases (MPs) are zinc-dependent endopeptidases, including matrix metalloproteinases (MMPs) and a disintegrin and metalloproteinases (ADAMs), implicated in various diseases such as cancer, neurodegenerative disorders, and cardiovascular conditions. Among MPs, ADAM-17, also known as tumor necrosis factor-α (TNF-α)-converting enzyme (TACE), plays a crucial role in extracellular matrix remodeling and cytokine release. Dysregulation of ADAM-17 contributes to inflammatory diseases, cancer progression, and immune modulation. While small-molecule inhibitors have been limited by off-target effects and instability, antibody-based approaches offer a more selective strategy. Monoclonal antibodies show promise in blocking ADAM-17 activity, but there are concerns about toxicity due to the lack of selectivity. Enhancing the binding affinity and selectivity of single-chain antibodies requires unraveling the structural details that drive MP targeting. This study uses yeast surface display (YSD) and fluorescence-activated cell sorting (FACS) to engineer single-chain variable fragment (scFv) antibodies with optimized complementarity-determining region 3 of the heavy chain (CDR-H3) conformations. Next-generation sequencing (NGS) was used to identify key residues contributing to high-affinity ADAM-17 binding. These findings offer a framework for designing monoclonal antibodies against ADAM-17 and other MPs, paving the way for novel antibody-based designer scaffolds with applications in developing therapeutics.

## 1. Introduction

Metalloproteinases (MPs) are a family of zinc-dependent endopeptidases, including matrix metalloproteinases (MMPs), and a disintegrin and metalloproteinases (ADAMs). These proteases are tightly associated with several diseases, including cancer, neurodegenerative and cardiovascular diseases, and gynecological disorders, when not properly regulated by their inhibitors and activators, making them attractive targets for developing novel protein-based therapeutics [1]. However, targeting MPs for therapeutic purposes has been particularly challenging due to their structural similarity, overlapping biological roles, and conserved catalytic domain, leading to off-target effects when broad-spectrum inhibitors are used [2,3]. For example, small molecules like Apratastat have shown limited success due to poor metabolic stability and adverse side effects [4,5].

Among MPs, a disintegrin and metalloproteinase-17 (ADAM-17), also known as tumor necrosis factor-α (TNF-α)-converting enzyme (TACE), is a key member of the MP family involved in extracellular matrix (ECM) remodeling and cytokine release. Dysregulation of ADAM-17 results in excessive TNF-α release, which is linked to inflammatory diseases, neurodegenerative disorders, fibrosis, cardiovascular dysfunction, and cancer [6]. Recent studies indicate that ADAM-17 also plays a role in immune modulation and tissue repair processes [7]. In the case of cancer progression, ADAM-17 facilitates the shedding of growth factors and cytokines that promote tumor growth and metastasis. For instance, in triple-negative breast cancer (TNBC), elevated ADAM-17 expression correlates with aggressive tumor behavior. Inhibiting ADAM-17 has been shown to reduce the proliferation and invasiveness of TNBC cells, suggesting that targeting ADAM-17 could be a promising strategy in cancer therapy [8]. Tissue Inhibitors of Metalloproteinases (TIMPs) are a family of endogenous proteins that play a vital role in regulating the activity of MPs [9]. TIMPs inhibit MPs by binding to their catalytic sites, thereby blocking enzymatic activity [10]. In humans, there are four recognized TIMPs. Each TIMP has a specific ability to inhibit different MPs. Among them, TIMP-3 is unique due to its strong binding affinity and enzyme inhibition for ADAM-17 [11]. 

Antibodies are emerging as powerful tools for selectively and efficiently targeting MPs, offering large binding interfaces, adaptable complementarity-determining regions (CDRs), and high-affinity binders [12,13,14]. Monoclonal antibodies (mAbs) that target ADAM-17 have shown promise in inhibiting tumor growth by preventing the shedding or release of key substrates, such as TNF-α and EGFR ligands, involved in cancer progression. For instance, MEDI3622, a potent and specific ADAM-17 inhibitory antibody, has demonstrated the ability to shrink tumors or stop their growth in models where tumors rely on EGFR, a key growth-signaling protein. However, its administration has been associated with toxicities similar to those observed by EGFR inhibitors, such as skin rashes, likely due to the disruption of normal EGFR signaling in healthy tissues [15]. Similarly, D1(A12), an IgG antibody targeting ADAM-17, has shown efficacy in reducing the shedding of substrates like TNF-α and EGFR ligands. However, detailed information about the potential side effects or challenges associated with D1(A12) is still limited [16]. Therefore, challenges such as off-target effects and achieving high affinity and specificity for ADAM-17 remain due to a lack of detailed sequence-level and structural insights, particularly in key interacting regions like the third complementarity-determining region of the heavy chain (CDR-H3).

Engineering antibodies against MPs has shown promise, especially when combined with directed evolution and display technologies such as yeast surface display [17,18,19,20,21]. Synthetic antibody libraries with diverse CDR-H3 regions, key determinants of antigen recognition, show promise in overcoming non-specific binding while achieving high-affinity interactions [22]. By precisely designing the amino acid sequence and structure of the CDR-H3 loop, single-chain variable fragment (scFv) antibodies with improved binding affinity and selectivity for specific targets can be generated. scFvs are often preferred over full-length antibodies in specific applications due to several advantages. Their smaller size (~27 kDa) allows for better tissue penetration, especially in solid tumors, compared to full-length antibodies (~150 kDa) [23]. scFv variants also lack the Fc region, reducing the risk of unwanted immune activation [24]. Furthermore, they can be efficiently produced in microbial systems like E. coli, lowering production costs compared to mammalian cell culture systems required for full-length antibodies [25]. This study aims to address whether structural insights can predict optimal CDR-H3 conformations for improved binding to ADAM-17, bridging the gap in sequence-level understanding. We employed yeast surface display (YSD) and fluorescence-activated cell sorting (FACS) to isolate scFv antibodies with higher affinity and improved selectivity for ADAM-17cd compared to the natural inhibitor TIMP-3. We conducted next-generation sequencing (NGS) to further identify critical residues and loop-length characteristics in CDR-H3 that enhance ADAM-17 binding. This analysis identified residues that enhance binding affinity and those that disrupt binding. Ultimately, we aim to establish a methodology that could be applied to other members of the MP family. By elucidating the determinants of engineered scFv antibodies to ADAM-17, our findings advance the development of efficient and specific binders and establish a framework for targeting other metalloproteinase family members.

## 2. Materials and Methods

### 2.1. Strain and Plasmid

Saccharomyces cerevisiae strains EBY100 (aGAL1-AGA1::URA3 ura3Δ52 trp1 leu2Δ200 pep4::HIS2 prb1Δ1.6R can1 GAL) and RJY100 were used for yeast surface display experiments [26]. The EBY100 strain was employed as the positive control for displaying TIMP-3, while the RJY100 strain was used to display single-chain variable fragment (scFv) variants of the naïve library on the yeast surface.

TIMP-3 was genetically fused to the N-terminus of the Aga2p protein and cloned into the pCHA vector backbone, ensuring a free N-terminus capable of binding to the ADAM-17 catalytic domain (ADAM-17cd). For scFv variants, the pCTCON2 vector was employed to express the scFv at the C-terminus of Aga2p [26].

### 2.2. Yeast Surface Display of TIMP-3 and scFv Antibodies

Yeast cells electroporated with display plasmid vectors were cultured overnight in minimal SDCAA medium (pH 6) at 30 °C and 250 rpm in a shaker incubator. The SDCAA medium was prepared with the following components: 20 g/L dextrose, 6.7 g/L yeast nitrogen base, 5 g/L Bacto Casamino Acids, 10.19 g/L sodium phosphate dibasic (Na_2_HPO_4_·7H_2_O), and 8.56 g/L sodium dihydrogen phosphate monohydrate (NaH_2_PO_4_·H_2_O). For protein expression, yeast cells were transferred to SGCAA medium (pH 6), which had the same components as SDCAA except that 20 g/L galactose replaced dextrose. The cultures were induced in SGCAA media for 18 h at 30 °C and 250 rpm, starting with an initial OD600 of 1.00. Yeast cells displaying TIMP-3 or scFv variants were then harvested at an OD600 of 0.2 and washed twice with 750 µL of ice-cold PBSA buffer (8 g/L NaCl, 0.2 g/L KCl, 1.44 g/L Na_2_HPO_4_, 0.24 g/L KH_2_PO_4_, 0.1% BSA, pH 7.4).

Next, the yeast cells were incubated with 6xHis-ADAM-17cd (Enzo Life Sciences, Farmingdale, NY, USA) or MMP-9cd auto-resistance cleavage (For selectivity assay) at a final concentration of 150 nM, based on previously established saturation conditions for TIMP-3 binding assays [17,27]. After incubation, cells were washed twice with 750 µL of ice-cold PBSA, and all subsequent steps were performed on ice. To detect surface expression and binding, the cells were first incubated with a primary mouse anti-c-Myc antibody (GenScript, Piscataway, NJ, USA) at a 1:100 dilution in PBSA (0.25 mg/mL) for 30 min on ice. Following two washes with ice-cold PBSA, the cells were incubated with antibodies: goat anti-mouse Alexa Fluor 488 antibody (2 mg/mL; Invitrogen, Carlsbad, CA, USA) and anti-6xHis monoclonal antibody conjugated with Alexa Fluor 647 (1 mg/mL; Invitrogen, Carlsbad, CA, USA). Both antibodies were used at a 1:100 dilution in PBSA, and the incubation was carried out for 30 min on ice, shielded from light. After final washes, the labeled cells were resuspended in 750 µL of PBSA for analysis using a BD Accuri™ C6 Plus flow cytometer (BD Biosciences, San Jose, CA, USA). Flow cytometry data were analyzed using FlowJo software (v10.10.0, FlowJo, LLC, Ashland, OR, USA).

### 2.3. High-Throughput Screening of the Yeast Surface Displayed scFv Library Using FACS

The scFv library was generously provided by the Wittrup lab at MIT’s Department of Chemical Engineering [26]. The library consisted of five variable heavy (VH) and three variable light (VL) chains linked by a glycine-rich peptide linker in a VL–VH orientation. To increase library diversity, mutagenesis was introduced in the heavy-chain complementarity-determining region 3 (CDR-H3), varying both loop length and amino acid composition [26]. The synthetic scFv library was recovered from glycerol stock by inoculating into 50 mL SDCAA medium (20 g/L glucose, 6.7 g/L yeast nitrogen base without amino acids, 5 g/L casamino acids, 10.4 g/L sodium citrate, 7.4 g/L citric acid monohydrate, pH 4.5 to discourage bacterial growth). After overnight growth at 30 °C in a shaker incubator, the culture was diluted into 500–1000 mL fresh SDCAA medium. For protein expression, yeast cells were transferred to SGCAA medium and grown until the culture reached an OD600 of 1.00 (approximately 10^7 cells/mL). yeast cells were incubated with the 6xHis-ADAM17-cd target, followed by primary and secondary antibody labeling as described above. The labeled cells were resuspended in ice-cold PBSA and sorted using a BD FACS Aria II cell sorter (BD Biosciences, San Jose, NJ, USA). A pentagonal gating strategy was applied to simultaneously detect Alexa Fluor-488 and Alexa Fluor-647 signals, representing positive expression and ADAM-17 binding, respectively. Additionally, a rectangular gate was used to isolate non-binding clones with positive expression signals but low Alexa Fluor-647 signals. The scFv library was subjected to two rounds of FACS. Two rounds of FACS were performed to enrich for high-affinity binders: the first round used 120 nM ADAM17cd, and the second round used 40 nM ADAM17cd to increase selection pressure for higher-affinity variants.

### 2.4. DNA Extraction for Sequencing and NGS Analysis

Plasmid DNA from isolated scFv variants was extracted using the Zymoprep Miniprep II kit (Zymo Research, Irvine, CA, USA). Extracted DNA was amplified by PCR, purified with the SV Gel and PCR Clean-Up System (Promega Corporation, Madison, WI, USA), and subjected to Sanger sequencing at Eurofins Genomics (Louisville, KY, USA).

For the next-generation sequencing (NGS) DNA analysis, plasmid DNA from the scFv antibody libraries—both positive and negative sorts—was extracted using the Zymoprep Miniprep II kit. To ensure high-quality sequencing, the extracted DNA was treated with Lambda Exonuclease to remove impurities, including yeast genomic DNA, following established protocols [28]. The variable heavy-chain genes were selectively amplified with Phusion high-fidelity DNA polymerase (New England Biolabs, Ipswich, MA, USA) using primers overlapping the CDR-H3 region. Amplified libraries were sequenced on an Illumina platform (Azenta/Genewiz, South Plainfield, NJ, USA).

DNA sequencing files in FASTQ format were processed by using Python 3.13.0 scripts to filter out low-quality DNA sequences, ensuring that only high-quality data was taken for further analysis. The filtered sequences were sorted by their frequency of occurrence to identify dominant clones or highly expressed genes in both final positive and negative binders’ libraries. Custom Python scripts were employed to analyze the CDR-H3 region, aligning the sequences and translating DNA into all six possible reading frames (three forward and three reverse) to accurately identify the correct protein-coding sequence. Mutations were detected by finding the CDR-H3 location on the gene, and further analysis revealed variations in amino acid composition and loop lengths within the CDR-H3 region. Finally, the frequency of amino acids at each position and the distribution of CDR-H3 lengths were quantified.

### 2.5. CDR-H3 Amino Acid Enrichment Analysis

To evaluate amino acid enrichment in the positive library relative to the negative library, CDR-H3 sequences of length seven were extracted from the NGS dataset. For each position (p) within the CDR-H3 loop, the frequency (F) of each amino acid (AA) was calculated separately for the positive and negative libraries using the formula:

F_AA,p = (count of AA at position p)/(total counts at position p).(1)

Enrichment ratios (E) were then determined by comparing amino acid frequencies in the positive library against those in the negative library:

E_AA,p = F_AA,p(+)/F_AA,p(−).(2)

An enrichment ratio greater than 1 indicated amino acid preference in the positive library, while a ratio less than 1 indicated relative depletion compared to the negative library.

### 2.6. Soluble Protein Expression and Purification

The pMopac16 periplasmic expression vector [21,29] was digested with the SfiI restriction enzyme, and the SynA2 antibody gene was inserted using the HiFi DNA Assembly kit (New England Biolabs). The resulting construct was verified by Sanger sequencing. Escherichia coli BL21 cells harboring the pMopac-SynA2 plasmid were grown by inoculating a single colony into 5 mL of 2 × YT medium supplemented with chloramphenicol and incubating overnight at 30 °C. The overnight culture (5 mL) was then transferred into 500 mL of fresh 2 × YT/chloramphenicol medium and incubated at 30 °C overnight without IPTG induction as previously discussed [30]. The following day, cells were harvested by centrifugation at 4500× *g* for 15 min at 4 °C, and the supernatant was discarded. The cell pellet was resuspended in a periplasmic extraction buffer (200 mM Tris-HCl (pH 8.0), 1 mM EDTA, 20% sucrose, and 50 μg/mL of lysozyme) at a volume of 3 mL per gram of pellet, mixed by vortexing, and incubated at room temperature for 5 min with gentle shaking. An equal volume of ice-cold double-distilled water was added, and the suspension was incubated on ice for 10 min with gentle shaking to induce osmotic shock. Cells were then centrifuged at 4500× *g* for 15 min at 4 °C, and the supernatant was collected and filtered through 0.45 μm membranes. The osmotic shock extract was purified using Ni-NTA affinity resin, with 20 mM imidazole in 25 mM sodium phosphate (pH 7.3), 300 mM NaCl as wash buffer, and 200 mM imidazole in the same buffer as elution buffer. Eluted fractions were concentrated using ultrafiltration units (10 kDa MWCO) by centrifugation at 4000× *g* for 15 min at 4 °C, followed by overnight dialysis at 4 °C against 50 mM HEPES and 150 mM NaCl, pH 7.3. Protein concentration was determined by spectrophotometry (NanoDrop, Thermo Fisher Scientific, Wilmington, DE, USA), and the purity of SynA2 was assessed by SDS-PAGE under reducing and non-reducing conditions. Final SynA2 preparations were stored in 10% glycerol at −80 °C.

### 2.7. Inhibition Study

The equilibrium inhibition constant (Ki) of SynAb-A2 toward ADAM17cd was determined using a protocol adapted for tight-binding inhibitors from previously established methods [17]. ADAM17cd (8 nM) was preincubated with SynAb-A2 (0.01–10 nM) in assay buffer (25 mM Tris, 2.5 µM ZnCl_2_, 0.005% Brij-35 [*w*/*v*], pH 9.0) for 1 h at 30 °C. No salt (CaCl_2_, NaCl, Na_2_SO_4_) was included in the assay buffer, as these ions inhibit TACE activity. The mixture was incubated for 1 h at 30 °C. After incubation, the fluorogenic substrate Mca-Pro-Leu-Ala-Gln-Ala-Val-Dpa-Arg-Ser-Ser-Ser-Arg-NH_2_ [Mca = (7-methoxycoumarin-4-yl)acetyl; Dpa = N-3-(2,4-dinitrophenyl)-L-α,β-diaminopropionyl] was added to a final concentration of 20 μM. Fluorescence was monitored using 340/30 nm excitation and 400/30 nm emission filters with a Synergy HT plate reader (BioTek, Winooski, VT, USA) at 37 °C. Measurements were taken every minute for 120 min, and initial reaction velocities were derived from the linear segments of the fluorescence traces. To determine Ki, the data were fitted using nonlinear regression to Morrison’s tight-binding inhibition model, with calculations based on a substrate Km of 7.76 μM. Analyses were performed with Prism 7 (GraphPad Software, San Diego, CA, USA). The reported Ki values represent the mean of two independent experiments; each performed in duplicate.

### 2.8. Training a Deep Learning Model for Predicting Binding to ADAM-17cd

Pre-trained Evolutionary Scale Modeling 2 (ESM-2) [31], with 36 transformer layers and 3 billion parameters, was used to extract zero-shot features from the CDR-H3 sequences for a total of 7789 positive binders and 9907 negative binders collected from high-throughput screening (80% for training and 20% for testing). Principal Component Analysis (PCA) was first applied to reduce feature dimensionality from 2560 to 217, retaining 95% variance. Then t-distributed Stochastic Neighbor Embedding (t-SNE) with a perplexity of 100 was performed on principal components to visualize features in two dimensions.

To examine whether providing additional local flanking sequences improved ESM-2 representations for binding prediction [32], the analyzed sequences were extended to include nine upstream (DTAVYYCAR) and 12 downstream (FDYWGQGTLVTVSS) amino acids flanking the variable CDR-H3 region. These flanking amino acid residues were identical across all sequences.

To further improve the performance of binding prediction, fine-tuning of protein language models was conducted beyond zero-shot features [33]. Sequences were split into training and testing sets, and the ESM-2 model (6B parameters) was fine-tuned in a two-step process. Firstly, Parameter Efficient Fine-Tuning (PEFT) using Quantized Low-Rank Adapters (QLoRA) [34] with a rank of 4 was applied to train the ESM-2 model on sequences with positive binding labels in the training set. A custom Masked Language Modeling (MLM) objective was used, where 15% of the CDR-H3 region was randomly masked, and the model was trained for six epochs using AdamW optimization with a learning rate of 5 × 10^−4^ to predict the masked amino acids. The upstream and downstream flanking sequences were not masked, as they were identical for all sequences, but were included to provide additional local context for the CDR-H3 region. Additionally, the sample loss was weighted by the sample weight, which was calculated as the number of times the specific CDR-H3 fragment was observed in positive binders obtained from the NGS data, divided by the total count of observed positive fragments. Secondly, a linear classifier was added and trained jointly with the last transformer layer of the ESM model using Supervised Fine-Tuning (SFT) with positive and negative labels (Figure S1).

## 3. Results

### 3.1. FACS-Based Screening of the Synthetic scFv Library Against ADAM17cd Resulted in Variants with Increased Expression and Enhanced Binding Compared to the Naïve Library

**Figure 1:**
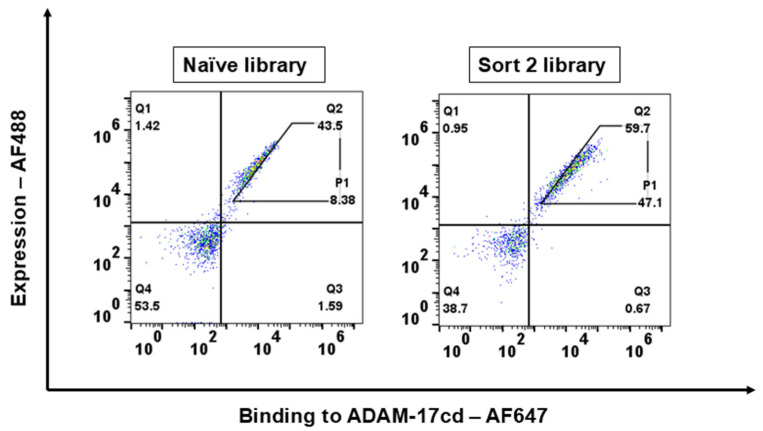
Analysis of scFv Libraries After Sequential FACS. Flow cytometry scatter plots showing the binding and expression profiles of the naïve and sort 2 positive scFv libraries towards ADAM-17cd. The left panel displays the naïve library, with gate (P1) marking cells that have both high binding and high expression levels. The right panel represents the Sort 2 library, showing an increased number of cells in the high-binding and high-expression gate (P1) compared to the naïve library.

The synthetic scFv library underwent two rounds of sequential FACS screening against ADAM-17cd, with decreasing concentrations in each round to enrich for cells displaying scFv variants with higher affinity. To assess the success of the enrichment process, both the final sorted positive population (Sort 2) and the naïve library were tested for binding to ADAM-17cd. Flow cytometry analysis used dual fluorescence labeling: Alexa Fluor-488 to assess scFv expression levels and Alexa Fluor-647 to quantify binding to ADAM-17cd. A significant shift in the binding signal was observed in the sorted library, with enhanced binding intensity. Specifically, the P1 gating region, representing yeast cells with high ADAM-17 binding affinity, exhibited approximately a 6-fold increase in population compared to the naïve library (Figure 1). These findings validate the sequential FACS screening strategy as an effective method for enriching scFv variants with enhanced expression and improved binding affinity for ADAM-17cd, supporting its effectiveness in generating high-affinity binders for metalloproteinase targets.

### 3.2. Positively Charged Residues Predominate in the Second Positive scFv Library Compared to Naïve and Negative Libraries: A Post-FACS NGS Perspective

**Figure 2:**
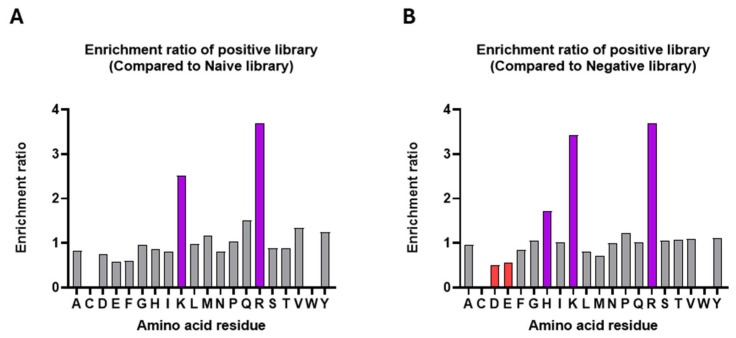
Amino Acid Enrichment in the Positive Library Compared to Naïve and Negative Libraries. (**A**) Enrichment of amino acids in the positive library relative to the naïve library. Significant enrichment is observed for positively charged residues such as lysine (K) and arginine (R). (**B**) Enrichment of amino acids in the positive library relative to the negative library. Positively charged residues, including arginine (R), lysine (K), and histidine (H), are enriched (highlighted in purple), while negatively charged residues like aspartic acid (D) and glutamic acid (E) are depleted (highlighted in red). Enrichment ratios were calculated as the frequency of each residue in the positive library divided by its frequency in the corresponding reference library.

Amino acid enrichment analysis of CDR-H3 sequences (Figure 2A) revealed a strong bias toward positively charged residues in the second-round positive scFv library compared to the naïve library. Lysine (K) and arginine (R) exhibited enrichment ratios near or above 3, indicating a strong preference for these positively charged residues. In contrast, negatively charged residues, such as aspartic acid (D) and glutamic acid (E), along with hydrophobic residues like phenylalanine (F), displayed enrichment ratios below 1, indicating a reduced presence in the positive library. In comparison, glycine (G) and serine (S) display enrichment ratios around 1, suggesting no significant preference for or against these residues in the positive binders. These results suggest that positively charged residues, particularly lysine (K) and arginine (R), are highly favored in the positive library, emphasizing their potential role in binding to ADAM-17. The strong enrichment of positively charged residues likely stems from electrostatic interactions with the negatively charged regions of ADAM-17cd, particularly its catalytic active site. Conversely, the depletion of negatively charged residues, such as aspartic acid (D) and glutamic acid (E), may reflect destabilizing or repulsive effects that impair binding. The neutral enrichment of glycine (G) and serine (S) suggests the necessity for these residues for maximal binding affinity through contributions to the structural flexibility of the CDR-H3 loop [35,36]. Comparison of the positive and negative libraries (Figure 2B) revealed a similar trend, with lysine, arginine, and histidine (H) enriched in the CDR-H3 motifs of positive binders. These residues may enhance affinity by promoting favorable electrostatic interactions with ADAM17cd. This pattern parallels previous reports for MMP-9 binders, where positively charged residues (K and R) were enriched and negatively charged residues (D and E) were depleted [22].

The enrichment of positively charged amino acids, such as arginine (R) and lysine (K), in antibody CDRs can significantly influence their binding interactions with target antigens. These residues can form electrostatic interactions, hydrogen bonds, and other non-covalent interactions with negatively charged or polar regions on the antigen, potentially enhancing binding affinity. However, an excessive positive charge can lead to poly-specificity, a phenomenon observed in synthetic antibody libraries, where a high density of R increases the risk of non-specific interactions with negatively charged surfaces [37]. Additionally, early-stage B-cell development is characterized by highly charged CDR-H3 loops, which may initially enhance binding but compromise specificity over time [38]. The synthetic library used in this study was pre-designed to minimize polyreactivity by reducing such residues and motifs. Additionally, sequence analysis of our isolated scFv variants using machine learning models trained on large deep sequencing data confirmed that they display low poly-reactivity (Figure S2), consistent with the design of the library that removed highly polyreactive motifs [39]. Therefore, achieving an optimal balance of positive charge is essential to maximize binding affinity while minimizing non-specific interactions, ensuring the development of high-affinity and selective scFv binders.

### 3.3. Enrichment Patterns of CDR-H3 Length: Short Loops in Negative Binders and Longer Loops in ADAM-17cd Binders

**Figure 3:**
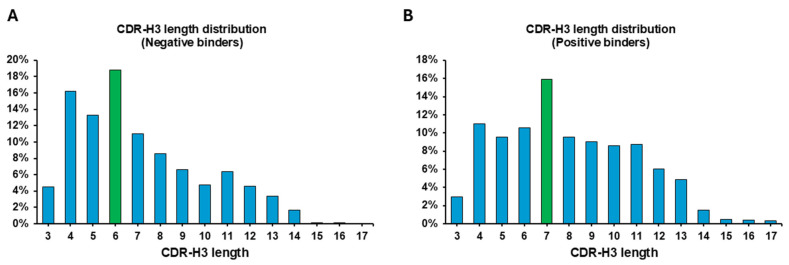
CDR-H3 Length Distribution in Negative and Positive Binders. Bar plots show the percentage distribution of CDR-H3 lengths among negative binders (**A**) and positive binders (**B**). (**A**) CDR-H3 length distribution in negative binders, with the majority of sequences having shorter lengths (e.g., 6 amino acids, highlighted in green). (**B**) CDR-H3 length distribution in positive binders, showing a broader range of lengths, with a peak at 7 amino acids (highlighted in green).

The relationship between the CDR-H3 loop’s length and binding affinity to ADAM-17cd reveals critical structural differences influencing effective interactions with the target. To explore this relationship, the length distribution of CDR-H3 sequences in both the negative and positive libraries was analyzed using next-generation sequencing (NGS) data. The results revealed a clear trend indicating that non-binders to ADAM-17 are predominantly enriched in shorter CDR-H3 length compared to binders (Figure 3A). The histogram demonstrates a decreasing frequency of sequences as their length increases for negative binders. Especially, a significant proportion of non-binders are concentrated at shorter lengths with a peak at 6 amino acids, suggesting a potential structural or functional limitation in their ability to interact with ADAM-17cd. In contrast, binders display a broader length distribution of sequence lengths, with a distinct peak at 7 amino acids (Figure 3B). This threshold suggests that CDR-H3 sequences shorter than 7 amino acids may lack the structural stability or key residues necessary for effective interactions with ADAM-17cd. A comparison with MMP-9 binders further supports the role of CDR-H3 length in target-specific binding preferences. Our previous study showed that MMP-9 binders predominantly feature longer CDR-H3 sequences, with 11 amino acids being the most enriched [22]. This length likely enables deeper penetration into the enzyme’s concave and uneven active site, facilitating critical interactions. In comparison, ADAM-17 binders show a preference for shorter CDR-H3 lengths, with a peak at 7 amino acids, which may be due to the relatively flatter binding interface of ADAM-17, particularly around its active site and adjacent structural regions. Despite some surface irregularities, ADAM-17’s binding site likely accommodates shorter CDR-H3 loops more effectively than MMP-9. These findings emphasize the role of CDR-H3 length as a key determinant of binding specificity and affinity, suggesting that optimal loop lengths are shaped by the structural topology of the target enzyme.

### 3.4. Amino Acid Enrichment and Positional Preferences in ADAM-17cd Positive Binders

The amino acid enrichment analysis was focused exclusively on CDR-H3 sequences of seven amino acids in length, as this was the most frequently observed length among scFv binders to ADAM-17. The enrichment trends in seven-residue CDR-H3 sequences from positive binders showed a pattern similar to that observed across the entire positive library when compared to the negative library. However, by focusing specifically on the seven-residue sequences, the analysis provided additional insights into specific residues that may contribute to binding affinity, beyond those identified in the broader library comparison.

**Figure 4:**
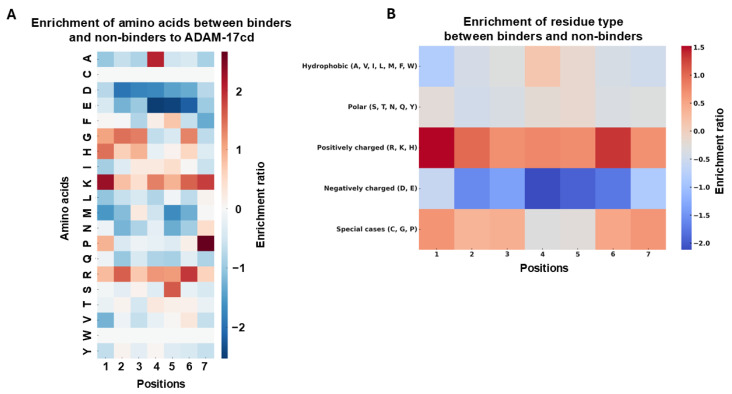
Enrichment of amino acids between positive binders and negative binders to ADAM-17cd. (**A**) Heatmaps showing the enrichment ratios of amino acids at each position (1–7) and (**B**) based on the type of residue in positive binders compared to negative binders. The color scale indicates enrichment ratios, with red shades showing positive enrichment (amino acids more frequent in binders) and blue shades showing negative enrichment (amino acids less frequent in binders). Significant enrichment is observed for positively charged amino acids like R and K at certain positions, while negatively charged and hydrophobic amino acids, such as D and E, are depleted.

The analysis revealed that positively charged residues were enriched, while negatively charged residues were depleted in the seven-residue CDR-H3 sequences of binders compared to non-binders (Figure 4A). Additionally, depletion of most hydrophobic residues is apparent across all positions (Figure 4B). Interestingly, special amino acids such as glycine (G), serine (S), and proline (P) are enriched at specific positions. These amino acids play distinct roles in shaping protein loops by balancing flexibility and rigidity. Glycine, the smallest amino acid, offers exceptional flexibility, allowing for sharp turns and dynamic regions in loops. Serine offers moderate flexibility while contributing stabilization through hydrogen bonding interactions. With its rigid cyclic structure, Proline introduces structural stability by locking parts of the loop into a fixed conformation [40]. These structural roles suggest that flexibility and stability must be balanced within the CDR-H3 loop to achieve optimal binding to ADAM-17cd. Together, the positional preferences of these amino acids appear to be strategically selected to maximize effective interactions with the ADAM-17cd, reinforcing the importance of loop conformation in antibody design.

### 3.5. The Engineered scFv Variants Demonstrated Enhanced Binding to ADAM-17cd Compared to TIMP-3

After two rounds of FACS enrichment, yeast clones isolated from the positive sort were evaluated for binding to ADAM17cd by flow cytometry. TIMP-3 was included as a positive control, as it is recognized as the strongest natural inhibitor of ADAM17 among the TIMP family [11]. Consistent with previous reports, TIMP-3 binds ADAM17 with sub-nanomolar affinity, with reported Ki values in the range of 0.2–0.5 nM [41,42,43]. In our assays, the engineered scFv variants exhibited stronger flow cytometry binding signals to ADAM17cd than TIMP-3, suggesting that the engineered antibodies can achieve enhanced apparent binding relative to the natural inhibitor.

**Figure 5:**
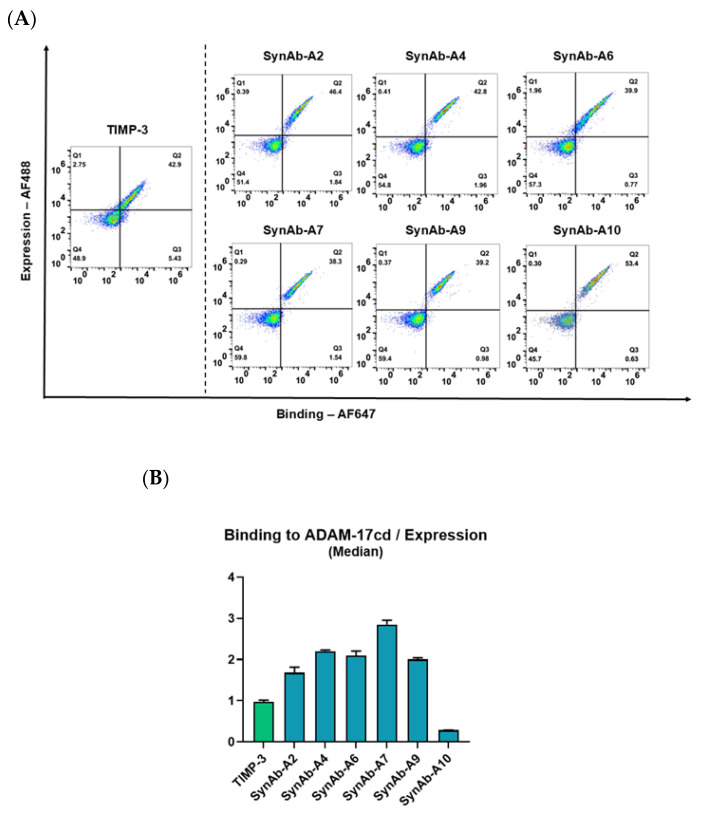
Binding to ADAM-17cd Normalized by Expression Levels (Median) for Isolated scFv Variants. (**A**) Flow cytometry scatter plots show isolated yeast-displayed scFv variants with enhanced binding activity to ADAM-17cd, using TIMP-3 as a positive control. The *x*-axis (APC channel) represents binding to 6xHis-ADAM-17cd (100 nM), while the *y*-axis (FITC channel) indicates scFv expression levels. (**B**) Bar plot showing the normalized binding of TIMP-3 and scFv variants to ADAM-17cd. Binding levels were normalized to expression (median values) to account for variability across samples. TIMP-3 (green) serves as positive control, representing the baseline for binding activity. The scFv variants exhibit a range of binding affinities, with SynAb-A7 showing the highest normalized binding and SynAb-A10 the lowest. Error bars indicate the standard deviation from three independent experiments.

Six unique scFv variants were isolated and identified. Median expression levels and binding intensities were quantified using a flow cytometry-based binding assay (Figure 5A). To ensure accuracy, the binding values were adjusted based on yeast surface expression levels. Since different scFv variants may be expressed at varying levels on the yeast surface, this normalization helped correct expression differences, ensuring that the binding signal reflected true binding affinity rather than variations in expression. The normalized binding signals of the scFv clones were then compared to TIMP-3, which served as a high-affinity reference. Notably, five out of the six scFv isolated clones demonstrated a higher binding per expression signal compared to TIMP-3 (Figure 5B). Among these, SynAb-A7 exhibited binding intensities up to three times higher than TIMP-3, indicating a significant improvement in binding affinity towards the target ADAM-17cd. Sequencing of the isolated scFvs revealed that the CDR-H3 regions of these clones contained 7, 9, and 11 amino acid residues. This finding also aligns with the CDR-H3 length distribution observed in the NGS analysis, where higher-affinity binders correlated with longer CDR-H3 loops (Table 1).

### 3.6. The Isolated scFv Clones Demonstrated Enhanced Selectivity for ADAM-17cd over MMP-9cd

**Figure 6:**
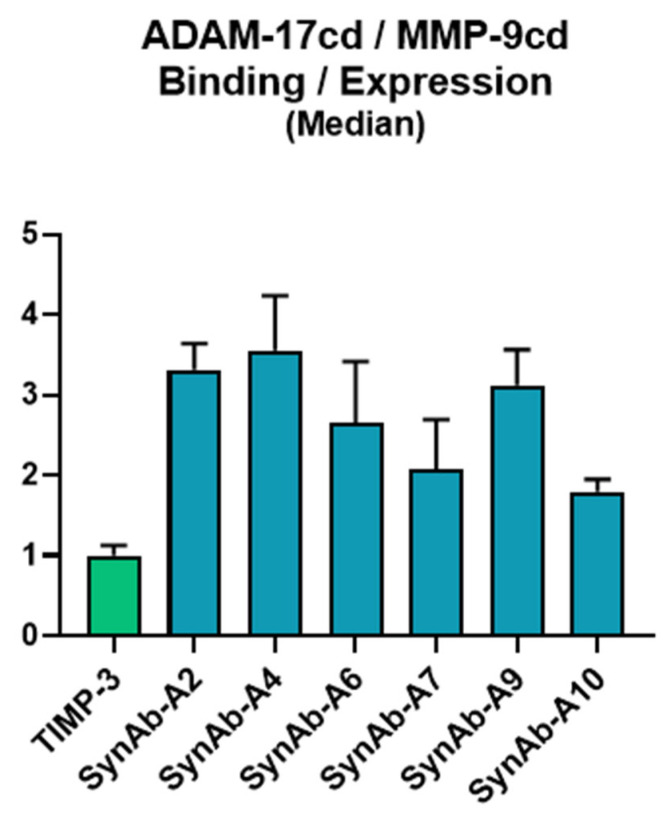
Binding selectivity of scFv variants towards ADAM-17cd and MMP-9cd. The plot illustrates the binding selectivity of yeast-displayed scFv variants, represented as the ratio of ADAM-17cd binding to expression divided by MMP-9cd binding to expression. Values are also normalized to TIMP-3 (shown in green), which serves as the positive control. Variations in binding selectivity highlight differences in scFv specificity for ADAM-17cd relative to MMP-9cd.

A binding assay on the yeast surface was performed to evaluate the selectivity of the isolated scFv clones towards ADAM-17cd. Although counter-selection FACS screening was not performed on this library, the scFv clones displayed improved selectivity for ADAM-17cd. The six scFv clones that exhibited a higher binding-to-expression ratio than TIMP-3 were tested against equal concentrations (100 nM) of both ADAM-17cd and MMP-9cd. The data were analyzed using the binding-to-expression ratio for ADAM-17cd, normalized against MMP-9cd binding, and further normalized to the positive control, TIMP-3. All tested clones exhibited greater selectivity for ADAM-17cd over TIMP-3, demonstrating an increase in selectivity of at least 2-fold (Figure 6).

**Figure 7:**
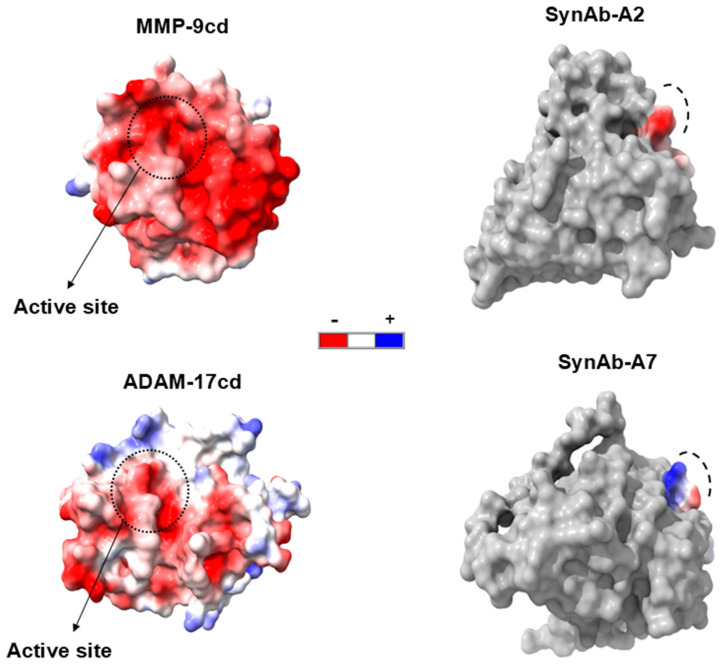
Analysis of CDR-H3 Surface Charge of isolated scFv variants. Surface charge distributions of the catalytic domains of ADAM-17cd and MMP-9cd are shown, focusing on their active sites. Both active sites exhibit a high density of negative charge, while the surrounding regions of ADAM-17 display more positive charge. Structural models of SynAb-A2 and SynAb-A7 highlight the CDR-H3 surface charge, with blue indicating positive charge and red indicating negative charge. Also, dashed lines mark the CDR-H3 region. scFv variant structures were modeled using AlphaFold3 with the same seed (42), while MMP-9cd and ADAM-17 structures were based on PDB entries 1GKC and 3CKI, respectively.

An improvement in binding was observed between SynAb-A2 and SynAb-A7 antibody variants. Both variants have seven amino acid residues in their CDR-H3 regions, and as previously discussed, SynAb-A7 exhibited the highest binding affinity signal to ADAM-17cd. However, SynAb-A7 also showed the lowest selectivity toward ADAM-17cd. Analysis of CDR-H3 amino acid composition revealed that SynAb-A7 variant possesses a predominantly positive charge, whereas SynAb-A2 has a more negatively charged distribution (Figure 7 and Figure S3). Examination of the catalytic domains of ADAM-17 and MMP-9 indicated distinct charge landscapes. The MMP-9 catalytic domain is predominantly negatively charged across both the active site and surrounding exosites. In contrast, the ADAM17 catalytic domain, although negatively charged at the active site, has neighboring regions enriched in positive charge. This difference in charge distribution likely influences the observed binding selectivity. Specifically, the positively charged CDR-H3 of SynAb-A7 can readily interact with the similarly structured active sites of both enzymes, leading to lower selectivity. In contrast, the negatively charged CDR-H3 of SynAb-A2 is repelled by the negative charge of the MMP-9cd but can interact favorably with the positively charged exosites or neighboring regions of ADAM-17cd. These findings highlight the critical role of CDR-H3 surface charge in modulating both binding affinity and specificity, providing insights into the design of selective scFv-based inhibitors for metalloproteinases.

### 3.7. Inhibition Assay of scFv SynA2 Reveals the Impact of CDR-H3 on ADAM-17 Binding and Inhibition

**Figure 8:**
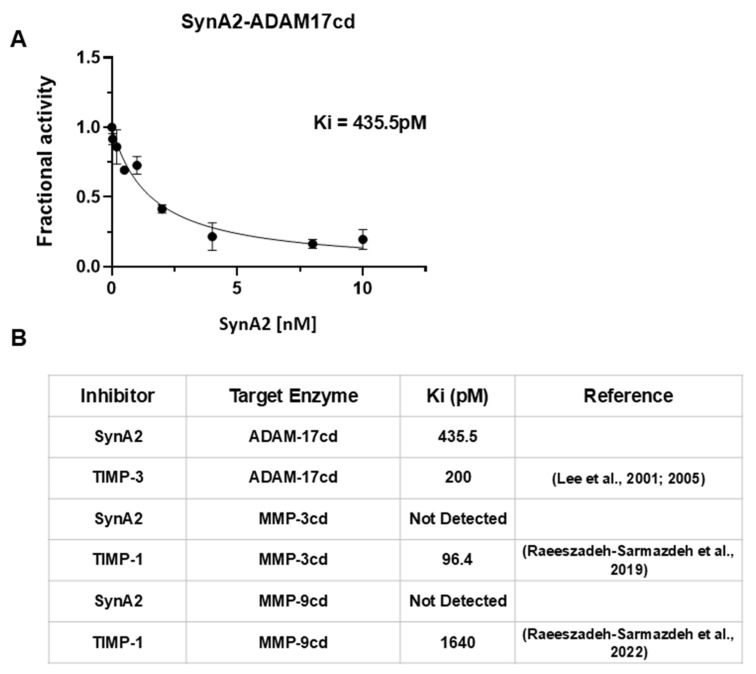
SynAb-A2 selectively inhibits ADAM17 but not MMP-3 or MMP-9. (**A**) ADAM17cd was incubated with SynA2 at various concentrations, followed by the addition of a fluorogenic substrate. The fluorescent signal generated upon substrate cleavage by ADAM17cd was recorded, and the reaction velocities were fitted to Morrison’s tight-binding equation to determine the Ki value, showing potent inhibition with a Ki of 435.5 pM. (**B**) Summary table of inhibition outcomes. TIMP-3 [44,46] and TIMP-1 [47,48] served as tight inhibitors of ADAM17, MMP-3, and MMP-9, respectively.

To extend our findings and quantitatively evaluate the inhibitory activity of SynA2 in solution, we cloned, expressed, and purified soluble SynA2 single chain antibody variant using an *E. coli* periplasmic expression system [19,21]. Inhibition of ADAM17cd, MMP-3cd, and MMP-9cd activity was assessed using a fluorometric substrate cleavage assay in the presence of increasing concentrations of the scFv. Reaction velocities were fitted to Morrison’s tight-binding equation to determine equilibrium inhibition constants (Ki). Consistent with the YSD results, SynA2 variant potently inhibited ADAM17cd with a sub-nanomolar Ki (435.5pM), while showing no detectable inhibition of either MMP-3cd or MMP-9cd even across a higher concentration range up to 1 μM (Figure 8). A broad range of SynA2 concentrations was incubated with 100 nM MMP-3 and 25 nM MMP-9 in enzyme inhibition assays, with MMP-3 concentrations varying from 15 to 2000 nM and MMP-9 concentrations from 0.4 to 100 nM. No significant inhibition of either enzyme was observed across these concentration ranges (Figure S4).

These inhibition studies, together with our binding data obtained from YSD, demonstrate that SynA2 is a highly potent and selective inhibitor of ADAM17, translating its binding specificity into enzymatic inhibition as well. Our findings are also in line with previous reports of selective protein inhibitors of ADAM17. For example, TIMP-3, the strongest natural ADAM17 inhibitor among the TIMP family, exhibits sub-nanomolar inhibition constants (200 pM) [44], while antibody inhibitors such as MEDI3622 (with 7 amino acid residues in CDR-H3) show high selectivity by potently blocking ADAM17-mediated substrate shedding without detectable activity against ADAM10, its closest homolog [45]. Together, these findings establish SynA2 as a potent and highly selective ADAM17 inhibitor. By combining strong inhibitory activity with a clear absence of off-target effects on MMP-3 and MMP-9, SynA2 emerges as a promising scaffold for therapeutic development, providing a functional validation of its selective binding properties.

### 3.8. Analysis of ADAM-17 Binding Using Protein Language Models

**Figure 9:**
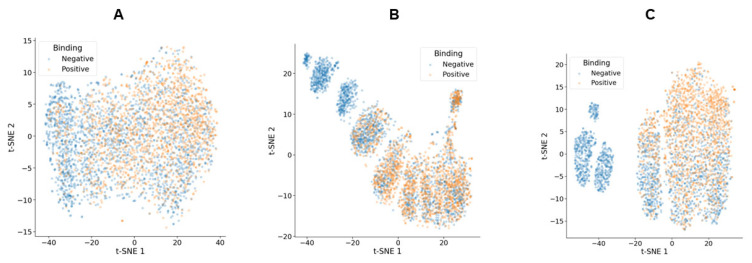
Representations from the ESM model. (**A**) Zero-shot representations from the ESM-2 model for CDR-H3 fragments of positive and negative binders in the test set. Some clustering is observed, but a significant overlap remains. (**B**) Zero-shot representations from the ESM-2 model for CDR-H3 fragments with upstream and downstream amino acids flanking CDR-H3. Some negative binders begin to form a distinct cluster, though positive binders are still intermingled with negative binders. (**C**) Representations from the fine-tuned ESM-2 model for CDR-H3 fragments of positive and negative binders in the test set. Positive binders in the upper right region show improved separation from negative binders, highlighting the effectiveness of the fine-tuning approach.

Initial predictions using ESM-2 demonstrated some ability to distinguish between ADAM-17 binder and non-binder scFv variants (Figure 9A). However, significant overlap between the two groups was observed, which limited classification accuracy. A linear classifier trained on the zero-shot representations from the training data yielded an out-of-sample F1 score of 0.723 for the binder class in the test set. This contrasts with previous studies for MMP-9, where zero-shot ESM-2 representations of the CDR-H3 region formed highly separable clusters, allowing for classification with high accuracy [22]. To enhance the model’s discriminatory power, upstream and downstream flanking sequences (although identical for all sequences) were incorporated, providing additional context that improved separation between binders and non-binders (Figure 9B). The F1 score of a linear classifier trained on zero-shot representations of CDR-H3 plus flanking regions improved to 0.73.

Further improvement was achieved through a two-step fine-tuning process, resulting in tighter clustering and reduced overlap between positive and negative binders (Figure 9C). The F1 score increased to 0.744, indicating enhanced classification accuracy. These findings suggest that the inclusion of flanking regions provides contextual information that enhances the model’s ability to differentiate between positive and negative binders.

## 4. Discussion

This study demonstrates the successful engineering of a single-chain variable fragment (scFv) antibody library with enhanced binding affinity and selectivity for ADAM17cd through yeast surface display, high-throughput FACS screening, and next-generation sequencing. By integrating structural analysis focused on CDR-H3, we identified key sequence and structural determinants of binding, providing insights into the design of high-affinity and selective scFv binders for ADAM17.

The CDR-H3 loop emerged as a key structural region driving both binding affinity and target selectivity. Seven-amino acid CDR-H3 loops were optimal for binding ADAM-17cd, suggesting a structural preference for the relatively flatter binding interface of ADAM-17cd. In contrast, shorter loops were predominantly enriched in the non-binding library, suggesting that structural limitations impede productive interactions. This observation aligns with the flatter yet uneven surface of ADAM-17cd, where shorter loops can achieve sufficient contact for binding. Conversely, MMP-9 features a deeper, more concave active site, favoring longer loops (~11 amino acids) for effective binding. This comparison highlights how loop length influences selectivity, underscoring the structural adaptability of scFv variants.

The amino acid composition of the CDR-H3 further revealed notable enrichment of positively charged residues, particularly lysine (K), arginine (R), and histidine (H), in high-affinity binders. These residues likely facilitate electrostatic interactions with the negatively charged active site of ADAM-17cd, enhancing binding affinity. However, our data also emphasizes the importance of balancing charge enrichment to maintain selectivity. For instance, SynAb-A7, which exhibited the highest binding affinity, also demonstrated lower selectivity, likely due to its positively charged CDR-H3 interacting with the densely negatively charged catalytic sites of both ADAM-17 and MMP-9. This electrostatic attraction may have contributed to non-specific binding across both targets. Conversely, SynAb-A2, featuring a negatively charged CDR-H3, exhibited greater selectivity for ADAM-17cd. This improved selectivity can be explained by favorable electrostatic interactions with the positively charged neighboring exo-sites of ADAM-17, while the negatively charged exosites of MMP-9 repelled such interactions. Functional inhibition studies also demonstrate that SynA2 is not only a high-affinity binder of ADAM17 but also a potent and selective inhibitor. In solution assays, SynA2 inhibited ADAM17 with sub-nanomolar inhibitory potency (Ki ≈ 435 pM), while showing no detectable inhibition of MMP-3 or MMP-9 even across the higher concentration range.

These findings suggest that optimal charge distribution within CDR-H3 can fine-tune binding selectivity. In summary, these findings provide a strong foundation for understanding protein–protein interactions targeting ADAM-17 and offer a framework for addressing similar challenges in other metalloproteinase family members.

## 5. Conclusions

In this work, we combined yeast surface display, deep sequencing, biochemical validations to engineer scFv antibodies with high affinity and selectivity toward ADAM-17cd. Our results demonstrate that CDR-H3 loop length and charge distribution are dominant determinants of both binding potency and target selectivity, with seven-residue loops and carefully balanced electrostatic profiles enabling optimal recognition of the ADAM-17 active and exosites. The discovery and functional validation of SynA2 as a sub-nanomolar, highly selective ADAM-17 inhibitor demonstrates that this platform can generate binders and inhibitors that rival natural inhibitors while minimizing cross-reactivity with related metalloproteinases. We also incorporated machine learning–guided predictions to identify ADAM-17 binders using high-throughput screening and deep sequencing data. The predictive precision was lower than that observed in our previous MMP-9 binding study [22], suggesting that the performance of such models is highly dependent on the quality, diversity, and sequence–function relationships of the underlying data. Together, these findings establish a generalizable platform for generating selective antibody-based inhibitors against metalloproteinases and other closely related MP families, supporting the development of precision therapeutics to overcome long-standing challenges of off-target activity.

## Figures and Tables

**Table 1:** Sanger sequencing of isolated scFv variants. The table presents the amino acid sequences of the light and heavy chains for each isolated scFv variant, with CDR-H3 highlighted in red.

SynAb	Light Chain	Heavy Chain
**A2**	**DIQMTQSPSSLSASVGDRVTITCRASQSISSYLNWYQQKPGKAPKLLIYAASSL** **QSGVPSRFSGSGSGTDFTLTISSLQPEDFATYYCQQSYSTPLTFGQGTKVEIKSGIL**	**EVQLVESGGGLVKPGGSLRLSCAASGFTFSNAWMSWVRQAPGKGLEWVGRI** **KSKTDGGTTDYAAPVKGRFTISRDDSKNTLYLQMNSLKTEDTAVYYCAR** **SYYEYVG** **FDYWGQGTLVTVSSGS**
**A4**	**EIVLTQSPGTLSLSPGERATLSCRASQSVSSSYLAWYQQKPGQAPRLLIYGASS** **RATGIPDRFSGSGSGTDFTLTISRLEPEDFAVYYCQQYGSSPSTFGPGTKVDIKSGIL**	**EVQLVESGGGLVKPGGSLRLSCAASGFTFSNAWMSWVRQAPGKGLEWVGRI** **KSKTDGGTTDYAAPVKGRFTISRDDSKNTLYLQMNSLKTEDTAVYYCAR** **PTSYYY** **FDYWGQGTLVTVSSGS**
**A6**	**DIQMTQSPSSLSASVGDRVTITCRASQSISSYLNWYQQKPGKAPKLLIYAASSL** **QSGVPSRFSGSGSGTDFTLTISSLQPEDFATYYCQQSYSTPLTFGQGTKVEIKSGIL**	**EVQLVQSGAEVKKPGESLKISCKGSGYSFTSYWIGWVRQMPGKGLEWMGIIY** **PGDSDTRYSPSFQGQVTISADKSISTAYLQWSSLKASDTAVYYCAR** **TDLHVHVYY** **FDYWGQGTLVTVSSGS**
**A7**	**DIQMTQSPSSLSASVGDRVTITCRASQSISSYLNWYQQKPGKAPKLLIYAASSL** **QSGVPSRFSGSGSGTDFTLTISSLQPEDFATYYCQQSYSTPLTFGQGTKVEIKSGIL**	**EVQLVQSGAEVKKPGESLKISCKGSGYSFTSYWIGWVRQMPGKGLEWMGIIY** **PGDSDTRYSPSFQGQVTISADKSISTAYLQWSSLKASDTAVYYCAR** **KKEYYSR** **FDYWGQGTLVTVSSGS**
**A9**	**DIQMTQSPSSLSASVGDRVTITCRASQSISSYLNWYQQKPGKAPKLLIYAASSL** **QSGVPSRFSGSGSGTDFTLTISSLQPEDFATYYCQQSYSTPLTFGQGTKVEIKSGIL**	**QLQLQESGPGLVKPSETLSLTCTVSGGSISSSSYYWGWIRQPPGKGLEWIGSIY** **YSGSTYYNPSLKSRVTISVDTSKNQFSLKLSSVTAADTAVYYCAR** **PGGYYDMEYLY** **FDYWGQGTLVTVSSGS**
**A10**	**EIVLTQSPGTLSLSPGERATLSCRASQSVSSSYLAWYQQKPGQAPRLLIYGASS** **RATGIPDRFSGSGSGTDFTLTISRLEPEDFAVYYCQQYGSSPSTFGPGTKVDIKSGIL**	**EVQLVESGGGLVKPGGSLRLSCAASGFTFSNAWMSWVRQAPGKGLEWVGRI** **KSKTDGGTTDYAAPVKGRFTISRDDSKNTLYLQMNSLKTEDTAVYYCAR** **TYASTPMLD** **FDYWGQGTLVTVSSGS**

## Data Availability

All data supporting the findings of this study are included within the article and its supplementary materials or available from the corresponding author upon reasonable request.

## Supplementary Materials

The following supporting information can be downloaded at: https://www.mdpi.com/article/10.3390/biom16010031/s1, Figure S1: Two-step Fine-tuning of ESM-2 flow chart. In step 1, a weighted Masked Language Modeling (MLM) objective is applied to the positive binders in the training dataset to fine-tune the ESM-2 representations. The sequences are extended to include upstream and downstream amino acids flanking the CDR-H3 region, as well as residues from the ADAM-17 active site, while masking is restricted to the CDR-H3 region. In step 2, a linear classifier is added and trained jointly with the last transformer layer of ESM-2 using supervised fine-tuning with both positive and negative labels; Figure S2: Comparing polyreactivity predictions of scFvs from different one-hot logistic regression models. The top panels show how scFv sequences with low (orange) and high (blue) polyreactivity are separated by model scores. The left side uses the model trained on the original FACS dataset, and the right side uses the model retrained on the larger high-throughput FACS dataset. The red line shows the score of the sequence being tested. The bottom panels compare model predictions (*x*-axis) with actual experimental polyreactivity measurements (*y*-axis). Sequences are grouped as high (orange), moderate (blue), or minimal (gray) polyreactivity. The red line again shows the score for the tested sequence. A high- and low-polyreactive sequence is shown here as a reference; Figure S3: Analysis of CDR-H3 Surface Charge of isolated scFv variants. Structural models of scFv variants illustrate the surface charge of CDR-H3, where blue represents a positive charge and red denotes a negative charge. The scFv variant structures were generated using AlphaFold3 with a consistent seed (42); Figure S4: SynA2 inhibition assay against MMP9cd and MMP3cd. (A) Enzyme inhibition assay of SynA2 with 25 nM MMP-9cd. SynA2 was tested at concentrations ranging from 0.4 to 100 nM. The fractional activity of MMP-9cd relative to the no-inhibitor control was fitted to the Morrison equation, and no detectable Ki was obtained. (B) Enzyme inhibition assay of SynA2 with 100 nM MMP-3cd. SynA2 was tested at concentrations ranging from 15 to 2000 nM. The fractional activity remained comparable to the uninhibited control across the entire range, confirming no detectable inhibition of MMP-3cd.

## Abbreviations

The following abbreviations are used in this manuscript:

MP	Metalloproteinase
MMP	Matrix Metalloproteinase
ADAM-17	A Disintegrin and Metalloproteinase-17
ECM	Extracellular Matrix
YSD	Yeast Surface Display
CDR-H3	Complementarity-Determining Region of the Heavy chain-3
FACS	Fluorescence-Activated Cell Sorting
scFv	Single-chain variable antibody fragment
TIMP	Tissue Inhibitors of Metalloproteinases
NGS	Next Generation Sequencing
MLM	Masked Language Model
ESM	Evolutionary Scale Modeling
t-SNE	t-distributed Stochastic Neighbor Embedding
mAb	Monoclonal antibody
PEFT	Parameter Efficient Fine-Tuning
QLoRA	Quantized Low-Rank Adapters
SFT	Supervised Fine-Tuning
cd	Catalytic domain
