# Supplementary material for: Engineering Single-Chain Antibody Fragment (scFv) Variants Targeting A Disintegrin and Metalloproteinase-17 (ADAM-17)
Source: Biomolecules. 2025 Dec 24;16(1):31. doi: 10.3390/biom16010031 (PMC12838881; doi:10.3390/biom16010031)
Supplement: Supplementary file 1 [file biomolecules-16-00031-s001.zip › biomolecules-3565196-supplementary.pdf]

Supplementary figures

Fig .S1

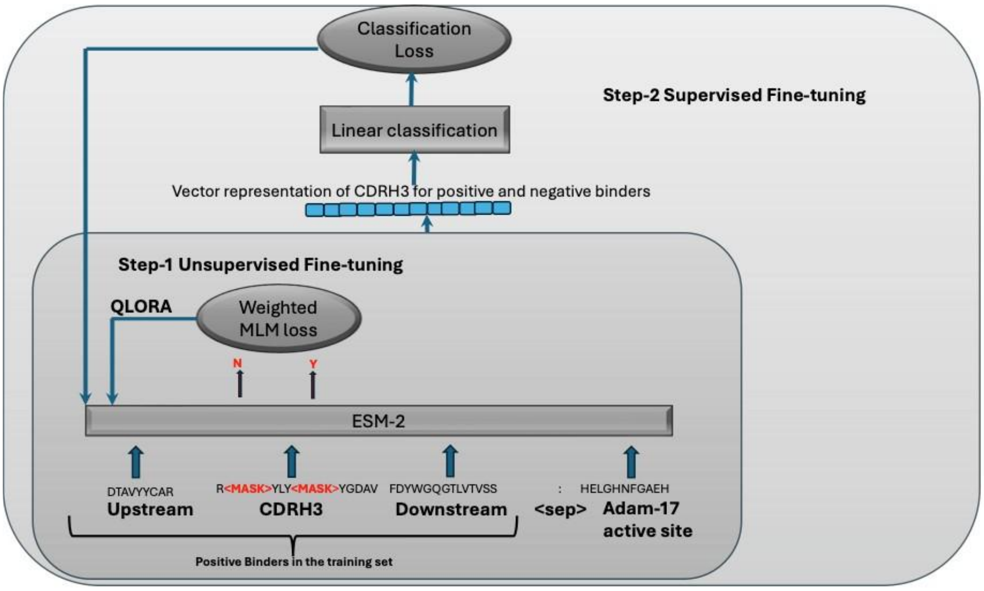

Figure S1. Two-step Fine-tuning of ESM-2 flow chart. In step 1, a weighted Masked Language Modeling (MLM) objective is applied to the positive binders in the training dataset to fine-tune the ESM-2 representations. The sequences are extended to include upstream and downstream amino acids flanking the CDR-H3 region, as well as residues from the ADAM-17 active site, while masking is restricted to the CDR-H3 region. In step 2, a linear classifier is added and trained jointly with the last transformer layer of ESM-2 using supervised fine-tuning with both positive and negative labels.

Fig .S2

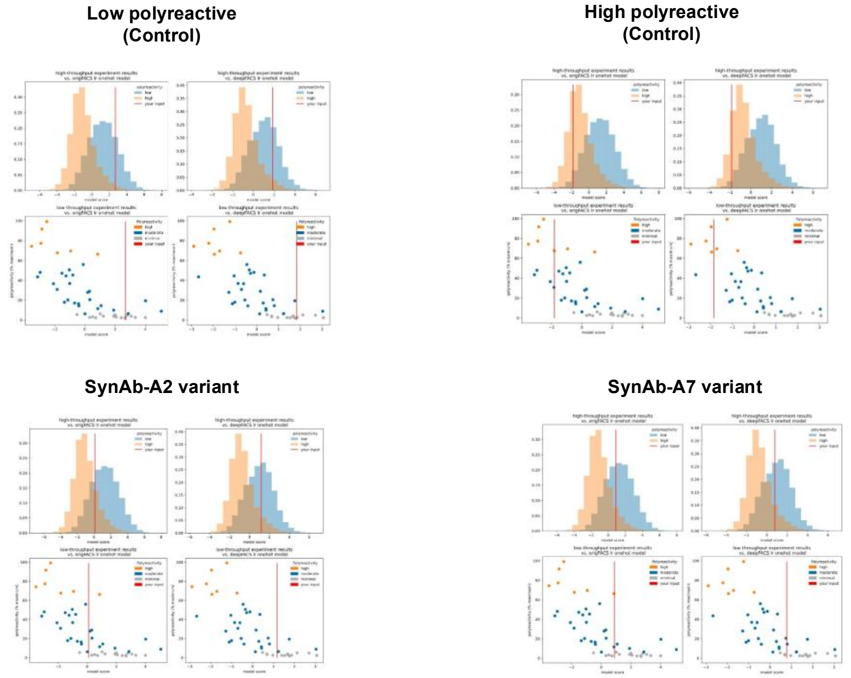

Figure S2. Comparing polyreactivity predictions of scFvs from different one-hot logistic regression models. The top panels show how scFv sequences with low (orange) and high (blue) polyreactivity are separated by model scores. The left side uses the model trained on the original FACS dataset, and the right side uses the model retrained on the larger high-throughput FACS dataset. The red line shows the score of the sequence being tested. The bottom panels compare model predictions (x-axis) with actual experimental polyreactivity measurements (y-axis). Sequences are grouped as high (orange), moderate (blue), or minimal (gray) polyreactivity. The red line again shows the score for the tested sequence. A high- and low-polyreactive sequence is shown here as a reference.

Fig .S3

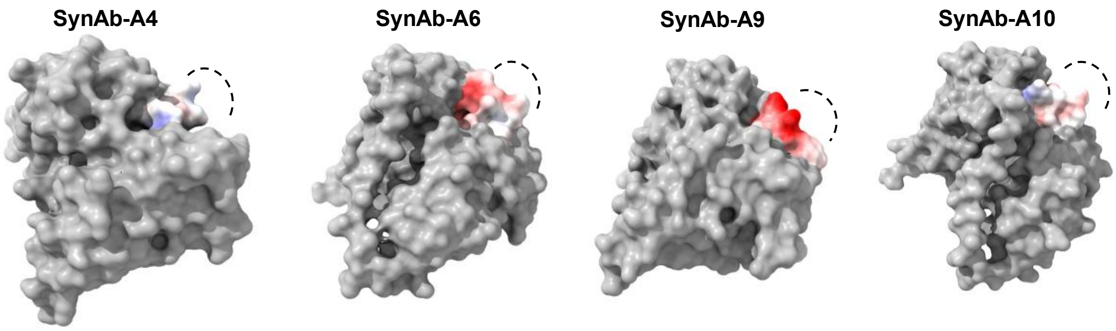

Figure S3. Analysis of CDR-H3 Surface Charge of Isolated scFv Variants. Structural models of scFv variants illustrate the surface charge of CDR-H3, where blue represents a positive charge and red denotes a negative charge. The

scFv variant structures were generated using AlphaFold3 with a consistent seed (42).

Fig .S4

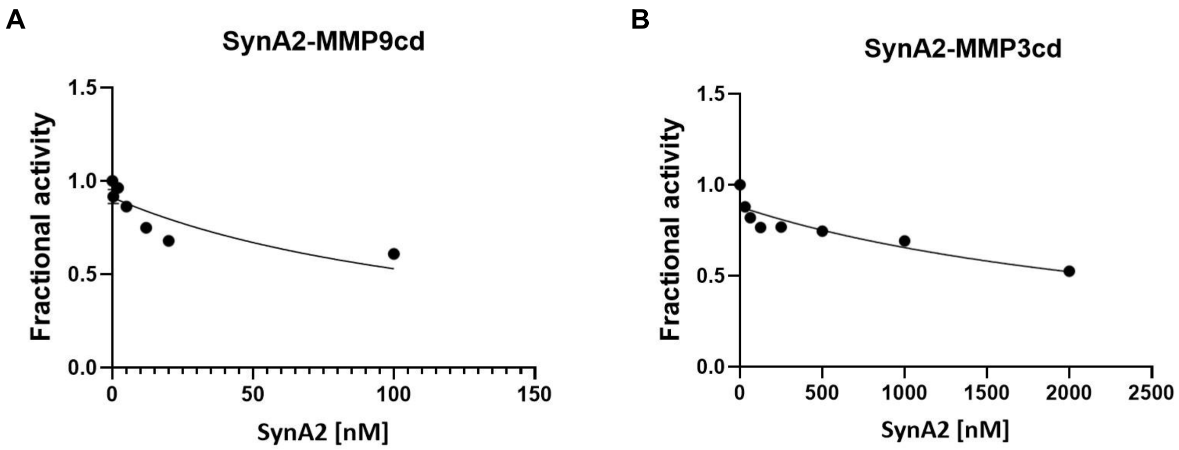

Figure S4. SynA2 inhibition assay against MMP9cd and MMP3cd. A) Enzyme inhibition assay of SynA2 with 25 nM MMP-9cd. SynA2 was tested at concentrations ranging from 0.4 to 100 nM. The fractional activity of MMP-9cd relative to the no-inhibitor control was fitted to the Morrison equation, and no detectable Ki was obtained. B) Enzyme inhibition assay of SynA2 with 100 nM MMP-3cd. SynA2 was tested at concentrations ranging from 15 to 2000 nM. The fractional activity remained comparable to the uninhibited control across the entire range, confirming no detectable inhibition of MMP-3cd.
